# Editorial: Meeting new challenges in translationally relevant neurodegenerative disease research

**DOI:** 10.3389/fnins.2024.1453770

**Published:** 2024-08-22

**Authors:** Caitlin Shannon Latimer, Zainuddin Quadri, David G. Cook

**Affiliations:** ^1^Department of Laboratory Medicine and Pathology, University of Washington, Seattle, WA, United States; ^2^Department of Physiology, University of Kentucky, Lexington, KY, United States; ^3^Geriatric Research Education and Clinical Center (GRECC), VA Puget Sound Health Care System, Seattle, WA, United States; ^4^Department of Medicine, Division of Gerontology and Geriatric Medicine, University of Washington, Seattle, WA, United States; ^5^Department of Psychiatry and Behavioral Sciences, University of Washington, Seattle, WA, United States

**Keywords:** neurodegeneration, models, neuropathology, genetics, translational research

Human neurodegenerative disease includes a broad range of complex disorders with distinct underlying pathologies and often overlapping clinical presentations that are driven by interactions between environment and genetic influences. Alzheimer's disease is the most common neurodegenerative disease, but Lewy body diseases and other synucleinopathies, Huntington's disease, motor neuron diseases, frontotemporal dementias, and many others, are important causes of cognitive and motor impairments that have limited available treatments. These disorders not only rob the individual of their independence but are also major stressors for caregivers and families and come with significant financial implications. It is estimated that 153 million people will be living with Alzheimer's disease (AD) by 2050, due to the world's aging population and the cost of dementia care is projected to increase from $1.33 trillion in 2020 to $9.12 trillion in 2050 (GBD 2019 Dementia Forecasting Collaborators). As such there is a great need and interest in better understanding, diagnosing, and treating these disorders. The landscape of neurodegenerative research is undergoing a significant transformation, fueled by rapid advancements in modern genetic and systems biology approaches.

Leading this revolution are discoveries in the genetics of neurodegenerative disease that reveal the intricate genetic architecture of these diseases. This includes rare, but highly penetrant causal mutations that have helped advance our understanding of the underlying mechanisms of disease. For example, familial forms of AD are due to mutations in genes involved in amyloid processing (Goate et al., 1991; Hardy, 2017), a discovery that has driven the development of biomarkers and targeted treatment strategies. The genetics of other neurodegenerative diseases have highlighted the complexity of the genetic underpinnings. For example, alterations in the Chromosome 9 Open Reading Frame 72 (C9orf72) gene emerged as a pivotal factor in the etiology of Frontotemporal Lobar Degeneration (FTLD) and Amyotrophic Lateral Sclerosis (ALS; Renton et al., 2011; Balendra and Isaacs, 2018). These two diseases have very different clinical presentations due to the selective vulnerability of disparate types of neurons, and yet the same hexanucleotide repeat expansion can cause either or both phenotypes, showcasing the interconnectedness of genetic variants across seemingly distinct conditions. Similarly, different mutations in a single gene, namely the microtubule-associated protein tau (MAPT) gene, can give rise to a spectrum tau pathology affecting different cell types (astrocytes, oligodendroglia, pyramidal neurons) and variable regional vulnerability (Giannini et al., 2022, 2023). The complexity of these disorders is further exemplified by the genetic heterogeneity of some neurodegenerative diseases, such as spinocerebellar ataxia (SCA), where multiple genes contribute to overlapping clinical symptoms and the loss of Purkinje cells remains a consistent hallmark, regardless of the causal gene (Klockgether et al., 2019).

In addition to these highly penetrant, yet rare causal genes, there have been significant advances in understanding the contribution of risk genes to the development of neurodegenerative disease. Sporadic, late onset AD in particular is likely driven by a combination of more common susceptibility genes (Jun et al., 2010; Pimenova et al., 2017). These include genes that result in an increased risk of AD, such as the APOE 4′ allele of the apoE gene as well as numerous protective variants that contribute to enhanced resistance and resilience to AD (Strittmatter et al., 1993; Kunkle et al., 2019). It has also become increasingly apparent that the impact of these various risk alleles is at least in part dependent on interactions with environmental exposures, including traumatic brain injury, air pollution, exercise, diet, and education (Gatz et al., 2006; Sakowski et al., 2024). The intricate interplay among genetic, cellular, and environmental factors underscores the multifaceted nature of neurodegeneration, and exploring these important yet complex interactions enriches our understanding of how they shape disease onset and progression.

Adding to the intricacy of neurodegenerative disease is our expanding appreciation for how many different factors contribute to the initiation and progression of the pathology and clinical expression of the disease. For example, biologic sex appears to underlie differences in susceptibility to neurodegenerative disease and response to treatment strategies (Arenaza-Urquijo et al., 2024). Additionally, while the pathologic proteins that in part define each of the neurodegenerative disease have long been the focus of diagnostic and therapeutic approaches, there are many other changes that occur and likely contribute to the disease process. These include alterations in inflammatory pathways and neuron-glial interactions (Chen et al., 2016; Patani et al., 2023; Castro-Gomez and Heneka, 2024), oxidative stress and mitochondrial dysfunction (Monzio Compagnoni et al., 2020), and vascular and white matter changes (Nasrabady et al., 2018; Paolini Paoletti et al., 2021). There exists a complex interplay between these various factors and the pathologic proteins that ultimately leads to neurodegeneration and clinical symptoms, but which aspects are the causal mechanisms vs. the downstream consequences of disease are still poorly understood and require continued investigation.

Amidst this complexity, the importance of integrating diverse approaches cannot be overstated. Animal models, bioinformatics, and human neuropathological studies play pivotal roles in bridging the gap between genetic discoveries and clinical realities, offering invaluable insights into disease pathogenesis and therapeutic strategies. The evaluation of the clinical and neuropathologic consequences of neurodegenerative diseases in humans is an essential component to neurodegenerative disease research that should not be overlooked. Most of these diseases are uniquely human, and models, while offering certain distinct advantages, are still just models that require integration with the evaluation of human central nervous system structure and function. The flip side is that human studies are inherently descriptive, necessitating the use of model systems to interrogate potential mechanisms for causal relationships with the pathology observed in the human disease. Therefore, a thoughtful, integrated approach that leverages both human tissues and data with appropriate model systems will yield the most translatable, potentially actionable results. Rodent models have dominated the field, largely driven by transgenic animals harboring rare, disease-causing mutations, which has limited the translatability of these models. However, newer models include the expression of more common, risk genes (Kotredes et al., 2021). Additionally, there has been an expansion in the use of animal models that naturally develop aspects of neurodegenerative disease; in particular dogs and non-human primates (Frye et al., 2021; Urfer et al., 2021; Cogram et al., 2024). While these large animal models are important for studying complex behaviors and brain-wide alterations, invertebrate systems, stem cells, and organoid models are ideal for mechanistic studies and high through-put screening assays (Giunti et al., 2021; Young and Goldstein, 2023; Pazzin et al., 2024). By harnessing the power of modern genetic and systems biology alongside interdisciplinary collaborations, we can unlock new avenues for translational research, ultimately paving the way for innovative therapies and personalized interventions tailored to the unique needs of patients.

In this Research Topic, we present this collection of articles that delve into the forefront of neurodegenerative disease research. Each article offers a unique perspective on the complexities of neurodegeneration and the promising avenues for future research, from elucidating novel genetic pathways underlying disease pathogenesis to exploring molecular mechanisms of resilience.

1. *Insights from C. elegans Models of ALS and FTLD-TDP* (Eck et al.). This review summarizes the insights gained from using the nematode *C. elegans* to study mechanisms of ALS and FTLD-TDP, emphasizing how this model can be leveraged to advance mechanistic research.
2. *Molecular basis of resilience to Alzheimer's disease* (Montine et al.). This review explores the cellular and molecular mechanisms underlying resilience to AD and the importance of studying human populations.
3. *Quantifying endo-lysosomal morphology in Alzheimer's disease* (Rose et al.). This study offers insights into endo-lysosomal dysfunction and cytopathology in sporadic AD, highlighting how human tissues can be leveraged to uncover potential disease mechanisms.
4. *Exploring brain extracellular matrix in age-associated neurodegeneration* (Hendrickson et al.). This perspective article investigates the translational potential of interregional variations in brain extracellular matrix components in age-associated neurodegenerative disorders, incorporating tissues from both human brain and animal models.

The future of neurodegenerative disease research depends on these types of integrated methods in order to effectively harness the power of human genetics, multiomics, neuropathology, bioinformatics, and animal models. Cross-disciplinary approaches in particular are necessary to ultimately unravel the complex pathways of neurodegeneration and identify novel diagnostic and treatment strategies for improving brain health and function.

## Author contributions

CL: Writing – review & editing, Writing – original draft. ZQ: Writing – review & editing. DC: Writing – review & editing, Conceptualization.
